# Self-perceived quality of health and satisfaction by elderly seen by the Family Health Strategy team

**DOI:** 10.1590/S1679-45082017AO3972

**Published:** 2017

**Authors:** Lilian Rigo, Raíssa Rigo Garbin, José Lucas Sani de Alcântara Rodrigues, Laerte Ribeiro Menezes-Júnior, Luiz Renato Paranhos, Cristiane Barelli

**Affiliations:** 1 Faculdade IMED de Passo Fundo, Passo Fundo, RS, Brazil.; 2 Universidade de Passo Fundo, Passo Fundo, Brazil.; 3 Universidade Estadual de Feira de Santana, Feira de Santana, BA, Brazil.; 4 Prefeitura Municipal de Itabaiana, Itabaiana, SE, Brazil.; 5 Universidade Federal de Sergipe, Lagarto, SE, Brazil.

**Keywords:** Health of the elderly, Chronic disease, Self care, Patient satisfaction, Family health strategy, Health services for the aged, Saúde do idoso, Doença crônica, Autocuidado, Satisfação do paciente, Estratégia saúde da família, Serviços de saúde para idosos

## Abstract

**Objective:**

To verify the profile of elderly followed up by Family Health Strategy teams and the influence of self-reported chronic diseases on demographic variables, describing self-perception and satisfaction with quality of health.

**Methods:**

This is a cross-sectional population- based study carried out with 301 elderly residents in two areas of a city in southern Brazil. Data were collected through a questionnaire based on the Health, Well-Being, and Aging Study adapted by researchers, and a playful scale to assess satisfaction with health. For the statistical analysis, the χ^2^ test was used (p<0.05).

**Results:**

Regarding cognitive assessment, the majority was classified as independent (86.4%), not requiring caregiver assistance to answer the questionnaire. The population was predominantly female (55.8%), White (64.8%), married (51.2%), and catholic (82.1%). A total of 47.5% considered their current health status as regular. Regarding satisfaction with health, 72.4% were happy, even reporting having at least one chronic health problem (58.5%).

**Conclusion:**

The presence of chronic diseases was reported by most respondents, and the practice of self-medication is significantly more frequent among the aged. Nevertheless, the degree of satisfaction with their health status was predominantly positive, both related to the previous year and compared to others of the same age.

## RESUMO

**Objetivo:**

Verificar o perfil de idosos acompanhados por equipes Estratégia Saúde da Família e a influência de doenças crônicas autorreferidas nas variáveis sociodemográficas, descrevendo a autopercepção e a satisfação com a qualidade de sua saúde.

**Métodos:**

Pesquisa transversal, de base populacional, realizada com 301 idosos residentes em duas áreas de um município do sul do Brasil. Os dados foram coletados por um questionário baseado no questionário do estudo Saúde, Bem-Estar e Envelhecimento, adaptado pelos pesquisadores, e um escala lúdica, para avaliar a satisfação com a saúde. Para a análise estatística, foi utilizado o teste χ^2^ (p<0,05).

**Resultados:**

Com relação à avaliação cognitiva, a maioria da população foi classificada como independente (86,4%), não necessitando de auxílio de cuidador para responder o questionário. A população foi predominantemente feminina (55,8%), branca (64,8%), casada (51,2%) e católica (82,1%). Ainda, 47,5% consideraram seu estado de saúde atual como regular. Com relação à satisfação com a saúde, 72,4% sentiam-se felizes, mesmo tendo relatado possuir, no mínimo, um problema de saúde crônico (58,5%).

**Conclusão:**

A presença de doenças crônicas foi relatada pela maioria dos entrevistados, com a prática de automedicação significativamente mais frequente nestes idosos. Apesar disto, o grau de satisfação com suas condições de saúde foi referido de forma predominantemente positiva, tanto em relação ao ano anterior quanto quando comparado a outras pessoas da mesma idade.

## INTRODUCTION

The visible process of aging of the world's population is linked to the increased longevity of the Brazilian population, and this occurs simultaneously with an increase in attention given to Public Health, which, in turn, goes through a solid movement of humanization and expansion of care.^(^ ^1^ ^)^ For the World Health Organization (WHO) there is no exact chronological age that defines a person as elderly. However, a large number of nations consider individuals aged 60 years or more are elderly, and in some developing or developed countries, this limit may be extended to 65 years.^(^ ^2^ ^)^ This population aging can be illustrated by the conformational change of the demographic pyramid of Brazil, demonstrating real rise in Brazilian longevity.^(^ ^3^ ^,^ ^4^ ^)^

The perception of the elderly regarding their age is surrounded by various factors that directly or indirectly affect quality of life. Practicing physical exercises, wellbeing and pleasant interaction among family members, as well as the circumstances of exerting their autonomy, are determining factors for personal satisfaction with own's health.^(^ ^5^ ^,^ ^6^ ^)^ The advancement in Brazil in care of the aged population, adding the implementations of new public policies destined to care for this portion of the population, influenced the production of new research geared towards this age range, especially on quality of life and aging.^(^ ^5^ ^,^ ^7^ ^)^ In this way, zeal with the elderly aims to maintain their health status and harmony with their family and the community, besides psychological and functional independence.^(^ ^8^ ^)^

Although many aged individuals do not associate personal satisfaction with health to absence of disease,^(^ ^5^ ^,^ ^7^ ^,^ ^8^ ^)^ one should consider how to promote their health, prevent diseases^(^ ^9^ ^)^ and remove avoidable causes of morbidity and mortality in order to afford a healthy and active aging.^(^ ^8^ ^,^ ^10^ ^,^ ^11^ ^)^ The program Family Health Strategy (ESF - *Estratégia Saúde da Família*) plays a fundamental role in the process of care of the elderly population,^(^ ^7^ ^,^ ^12^ ^)^ because if an individual is unable to move and go to the health service facilities due to some deficiency, the ESF provides external activities, guaranteeing health and quality of life to this population.^(^ ^12^ ^,^ ^13^ ^)^ Moreover, these individuals should not be addressed in a fragmented manner, *i.e.,* isolated from their family and social contexts, as well as their values. The program should develop new humanized actions based on intersectoral interactions, which are technically competent and socially appropriate.^(^ ^14^ ^,^ ^15^ ^)^ Understanding what the elderly value, and how they experience their health issues, social relationships, family interactions, leisure, work and access to their basic needs, can orient health promotion processes of the teams, helping the current and future elderly to live this stage, and implementing actions that bring about changes in the healthcare system in Brazil as provided in public policies.

## OBJECTIVE

To verify the profile of the elderly followed by Family Health Strategy Teams and the influence of self-reported chronic diseases on sociodemographic variables, besides describing self-perception of their quality of life and their satisfaction with it.

## METHODS

This study was approved by the Ethics and Human Research Committee of the *Universidade de Passo Fundo* (UPF), under number 438/2010 and CAAE: 0264.0.398.398-10.

This is a quantitative observational cross-sectional study. The study population comprised elderly residents in the areas covered by the neighborhoods of Adolfo Groth and Planaltina, in the city of Passo Fundo (RS), during the period of August to December 2011.

The study was carried out in two Primary Care Units (PCU) with the ESF of Passo Fundo (RS), defined by convenience and for having teams of the (PET - *Programa de Educação pelo Trabalho para Saúde*) [Work Health Education Program]. This city is situated in the northern region of the state of Rio Grande do Sul, and according to the census of the Brazilian Institute of Geography and Statistics (IBGE - *Instituto Brasileiro de Geografia e Estatística*) of 2007, has a population of 183 thousand inhabitants, with an area corresponding to 789km^2^. Its healthcare system has four hospitals, 23 outpatient clinics, and 15 ESF teams. The ESF implementation process in the city has increased, having been initiated in the year 2000 with four teams and 7.7% coverage of the population. In 2003, three more teams were implemented, reaching coverage of 13.4%; and, in 2004, another eight teams started activities. Currently, there are 15 teams, with 28.8% coverage of the population, according to the Primary Care Information System (SIAB) data from 2008, provided by the Municipal Department of Health.

Based on the data entered into SIAB in two ESF covered by the study, the total population of residents was 9,000 people, and roughly 15% of them were aged over 60 years, totaling 1,350 individuals. The following parameters were adopted to calculate the target population: 5% sampling error, 95% confidence interval, and 40% maximum prevalence. Another 10% were added to cover potential losses and/or refusals. Since the elderly population for the two healthcare teams was 1,500 individuals, 338 people would be a part of the study.

As inclusion criteria, the subjects should be aged 60 or more years;^(^ ^2^ ^)^ reside in the city of Passo Fundo (RS); be able to communicate and coherently answer the questions included in the interviewer's instruments. Excluded were those subjects that changed residence during the home visits for data collection.

Data was collected by two previously trained undergraduate medical students at *Universidade Federal de Sergipe* (UFS), participants of the PET - Saúde Program in two primary care units selected for the study. The instruments were calibrated for application of questionnaires during data collection. The elderly were approached by means of home visits and also in the waiting room of the primary care unit. For quality control of the study, the data collection team was trained based on validated and reliable instruments (questionnaires). A pilot test was conducted on 10% of sample to verify the proposed methodology.

To characterize the studied population according to the sociodemographic profile, perception and satisfaction with their health, a structured questionnaire was used. It was adapted by the researchers, based on the social and health questionnaire of the Health, Well-being, and Aging Study, (SABE - *Estudo Saúde, Bem-estar e Envelhecimento*) available at http://www.fsp.usp.br/sabe.^(^ ^16^ ^)^ A few questions were removed from SABE related to personal data (sex, age, ethnic group, marital status, and religion), living conditions (number of residents per household, type of house, building material of the house, provision of water, electricity, living alone, receiving help from someone else/help from others living in the same house, and help from any institution over the last year), and health status (current health status, perception of current health status, how your health was one year ago, comparing your health with people of the same age, degree of satisfaction with your health, references to chronic diseases, quantity of reported chronic diseases, and chronic use of medications).

The cognitive assessment in SABE, conducted by means of the Pfeffer questionnaire, was applied to the elderly at the beginning of data collection. Such questionnaire is used in medical practice to evaluate cognitive impairment of individuals in their activities, classifying them as independent or dependent in daily life activities. Based on the results, the data were informed by the participants or with the help of caretakers. The Pfeffer questionnaire comprises ten items related to the individual's capacity to carry out Activities of Daily Lving (ADL) and sociocognitive functions, such as making purchases, preparing meals, keeping up with current events, paying attention to radio and television programs, and being able to discuss them. The lower the score obtained by the individual, the higher their level of independence and autonomy. The minimum score is zero, and the maximum is 30.^(^ ^17^ ^)^

The questionnaire includes seven faces drawn (with expressions, such as one neutral, three discouraged, and three motivated faces) and was utilized as a playful instrument to measure satisfaction with health of the elderly. This instrument was adapted from another study^(^ ^10^ ^)^ conducted with an aged population to verify life satisfaction. In this way, the elderly were asked only about degree of satisfaction relative to their health, measured on a scale from 1 to 7, using visual recognition.

In order to evaluate the instruments to be used in the research, a pilot test was performed and served to train the investigators and adjust the questions to the subjects. It had been applied to 10% of the established sample (30 elderly people).

The descriptive variables were distributed into two groups: (1) sociodemographic variables, composed of age range, site of the ESF, ethnic group, marital status, religion, residents per household, type of house, building material used in the house, water supply, electricity in the house, if living alone, if receiving help from another, if receiving help from someone living in the same house, and if received help from an institution over the last year; (2) variables related to self-perception and satisfaction with health, addressing the current health status, how subjects currently evaluated their health, how health was one year before, comparison of health with other people of the same age, degree of satisfaction with one's own health, and chronic use of medications.

The dependent variable used was reported chronic diseases, obtained among those who reported having or not having chronic diseases. Chronic diseases were measured according to the self-reported answers regarding the presence of one of the following chronic diseases: hypertension; *diabetes mellitus;* cardiac diseases (angina, infarction, and other heart problems); pulmonary diseases (bronchitis, asthma, and/or emphysema); cancer, and arthropathy (arthrosis, arthritis, and/or rheumatism).

The variables selected to test the associations with the sociodemographic variables were age range, site of the ESF, ethnic group, marital status, religion, number of residents per household, type of house, if living alone, and if receiving help from another; and variables of self-perception of health, namely, chronic use of medications.

The quantitative data were encoded and entered into a specific database for descriptive and inferential statistical analysis, and subsequently exported to the Statistical Package for the Social Sciences (SPSS) software, version 17.0, for processing. To assess the association between the dependent variable (reported chronic diseases) and the independent variables, Pearson χ^2^ test was used, with a 5% significance level, and a 95% confidence interval. The variables associated with the outcome were those with a significance ≤0.05.

## RESULTS

In addition to the eligibility criteria, 37 subjects did not appropriately answer all questions of the study, with a loss of 11% of sample. Therefore, the final sample of the study was composed of 301 individuals.

As to the cognitive evaluation performed at the beginning of the study via the Pfeffer questionnaire, most of the elderly were classified as independent (85.4%), with no need of the help from a caregiver to answer the questionnaire. Some individuals required the caretaker assistance (15.6%).

The results of the sociodemographic variables of the elderly evaluated showed that 168 were female, with a mean age of 69.93 (±7.33) years, a minimum of 60 years and a maximum of 90 years (Table 1).

**Table 1:** Demographic variables of the elderly

Demographic variables		n (%) (n=301)
Age range, years		
	60-70	167 (55.5)
	71-80	103 (34.2)
	81-90	30 (10.0)
	Over 91	1 (0.33)
Ethnic group		
	White	192 (64.8)
	Asian	2 (0.7)
	Indigenous	3 (1.0)
	Mulatto	80 (26.6)
	Black	19 (6.3)
	Losses	2 (0.66)
Marital status		
	Divorced	16 (5.3)
	Separated	21 (7.0)
	Widow(ed)	80 (26.6)
	Married	154 (51.2)
	Cohabiting	12 (4.0)
	Single	3 (1.0)
	Losses	15 (4.9)
Religion		
	Catholic	247 (82.1)
	Protestant	3 (1.0)
	Evangelical	41 (13.6)
	Spiritist	2 (0.7)
	Budist	1 (0.3)
	Other	4(1.3)
	None	3 (1.0)
Residents per household		
	1	44 (14.6)
	2	119 (39.5)
	3	64 (21.3)
	4	45 (15.0)
	5	10 (3.3)
	6	19 (6.3)
Type of house		
	Own	280 (93.0)
	Rented	15(5.0)
	Donated or borrowed	6 (2.0)
Building material of the house		
	Wood	76 (25.2)
	Brick/masonry	156 (51.8)
	Mixed	69 (22.9)
Water supplied by the sewage company		
	Yes	298 (99.0)
	No	3 (1.0)
Electricity		
	Yes	301(100)
	No	0 (0)
Living alone		
	Yes	62 (20.6)
	No	236 (78.4)
	Did not answer	2 (0.7)
	Losses	1 (0.3)
Receiving help from another		
	Yes	201 (66.8)
	No	79 (26.2)
	Losses	21 (7)
Help from someone living in the same house		
	Yes	174 (57.8)
	No	88 (29.2)
	Did not answer	12 (4.0)
	Losses	27 (9)
Help from someone not living in the same house		
	Yes	80 (26.6)
	No	172 (57.1)
	Losses	47 (10.6)
Help from an institution over the last year		
	Yes	17(5.6)
	No	241 (80)
	Did not answer	20 (92.4)
	Losses	23(7.6)

As to health conditions, 47.5% of elderly considered their current health status as regular, and most of them considered their current health similar to that of one year before (63.5%) and to people of the same age (44.5%). Regarding chronic diseases, 90.7% referred to some chronic disease, and 70.4% reported the chronic use of medication (Table 2).

**Table 2:** Variables of self-perception about quality and satisfaction with their health

Variable		n (%) (n=301)
Current health status		
	Very good	12 (4.0)
	Good	121 (40.2)
	Regular	143 (47.5)
	Poor	22(7.3)
	Very poor	3(1.0)
How do you consider your current health status		
	Good	133 (44.2)
	Regular	143 (47.5)
	Poor	25 (8.3)
One year ago, your health was		
	Better	49 (16.3)
	The same	191 (63.5)
	Worse	61 (20.0)
Comparing your health with other people of the same age, yours is		
	Better	123 (40.9)
	The same	134 (44.5)
	Worse	44 (14.6)
Degree of satisfaction with your health - playful scale		
	3 sad faces	1 (0.3)
	2 sad faces	4(1.3)
	1 sad face	10 (3.3)
	1 indifferent face	68 (22.6)
	1 happy face	69 (22.9)
	2 happy faces	68 (22.6)
	3 happy faces	81 (26.9)
Playful scale classification		
	Happy	218 (72.4)
	Unhappy	83 (27.6)
References to chronic diseases		
	Yes	273 (90.7)
	No	24 (8.0)
Quantity of chronic diseases reported		
	0	125 (41.5)
	1	85 (28.2)
	2	51 (16.9)
	3	31 (10.3)
	4	5 (1.7)
	5	4(1.3)
Chronic use of medication		
	Yes	212 (70.4)
	No	89 (29.5)

Table 2 further shows the degree of satisfaction with their health using the playful scale, presence of chronic diseases, and use of medication. For the classification of satisfaction with one's health, all the sad and indifferent choices were pooled as “not happy” (the neutral face and the three discouraged faces) and all the choices of the happy faces as “happy” (the three motivated faces). Hence 218 elderly (72.4%) felt happy with their health.

The bivariate analysis of table 3 displays the reported chronic diseases and their relation with age, sex, ESF and chronic use of medications. The results showed that most of the individuals who had reported chronic diseases were on continuous use medications (86%), and the relation between these variables was statistically significant (p<0.001). The remaining independent variables (sociodemographic) examined in the analyses did not present statistical association (p>0.05).

**Table 3:** Inferential analysis of the independent variables and presence of reported chronic diseases

Variables		Reported chronic diseases			
		No n (%)	Yes n (%)	Total n (%)	p value
Age range					>0.005
	60-70	17 (70.8)	148 (54.2)	165 (62.5)	
	Over 70	7 (29.1)	129 (45.7)	136 (37.4)	
Sex					>0.005
	Male	13 (54.1)	116 (42.4)	129(43.4	
	Female	11 (45.8)	161 (57.6)	172 (56.6)	
ESF					>0.005
	Adolfo Groth	10(41.6)	138 (50.6)	148 (49.8)	
	Planaltina	14 (58.3)	139 (49.4)	153 (51.1)	
Chronic use of medication					*<0.001
	Yes	0	212 (86.0)	212 (79.1)	
	No	24 (100.0)	65 (13.0)	89 (20.9)	
Ethnicity					0.470
	White	15(62.5)	181 (65.3)	196 (65.1)	
	Black, “Negro”, Indigenous, and Asian	9 (37.5)	96 (34.7)	105 (34.9)	
Marital status					
	Married	11 (45.8)	143(51.6)	154 (51.2)	0.370
	Separated, divorced, widow (ed) and single	13 (54.2)	134 (48.4)	147 (48.8)	
Religion					0.240
	Catholic	18 (75.0)	229 (82.7)	247 (82.1)	
	Protestant, Evangelical, Spiritist, Budist	6 (25.0)	48 (17.3)	54 (17.9)	
Residents per household					0.371
	1-3	17 (70.8)	210 (75.8)	227 (75.4)	
	4-6	7 (29.2)	67(21.2)	74 (24.6)	
Type of house					0.515
	Own	22(91.7)	258 (93.1)	280 (93.0)	
	Rented, donated, or borrowed	2 (8.3)	19 (6.9)	21 (7.0)	
Live alone					0.241
	Yes	7 (29.2)	58 (20.9)	65(21.6)	
	No	219 (79.1)	17 (70.8)	236 (78.4)	
Receives help from someone					0.398
	Yes	15(62.5)	186 (67.1)	201 (66.8)	
	No	9 (37.5)	91 (33.9)	100 (33.2)	

* p<0.05.

Statistically significant relation.

ESF: Family Health Strategy program.

## DISCUSSION

The subjectivity of the concepts of quality of life, as well as of health and aging, suffers the interference of various factors inherent to human beings, such as physical skills and abilities, psychological conditions, social interactions, and environment. The concept of quality of life related to health established the study of these factors for those intimately involved with the individual's physical, psychic, and social condition.^(^ ^18^ ^-^ ^19^ ^)^

The profile of the aged population found in this study was mostly White women, living in their own homes and generally accompanied by someone. Very similar profiles were found in studies by other authors.^(^ ^18^ ^,^ ^19^ ^)^ Alves et al.,^(^ ^20^ ^)^ in a cross-sectional study of data obtained from the IBGE, evaluated the factors associated with the functional disability of the elderly, and obtained a profile characteristically similar to that of this study. Their sample was mostly White females living in urban areas, and living accompanied by someone else. They also stated quality of health was directly related to the functional independence of the elderly. Independence and autonomy of the aged are decisive factors to modify self-perception relative to one's health.^(^ ^5^ ^,^ ^11^ ^)^ However, as one could observe, many elderly still lived with their families, which could infer a relationship of semi-dependence.

As to the proportional distribution of the senior citizens residing in the areas of both ESF units in the city of Passo Fundo (RS), the number of individuals by age range – 60 to 69 years, was in accordance with other studies carried out on the profile of the elderly in other cities, both in and out of the State of Rio Grande do Sul.^(^ ^18^ ^-^ ^20^ ^)^

The method to choose the sample may present limitations as to generalization, but the authors considered that it satisfactorily represented the opinion of the aged people investigated, since it used a sample calculation of the study population.

The proportion of the elderly that consider their current health status as good and/or regular was an important result of this study (91.7%). Xavier et al.^(^ ^21^ ^)^ used a similar methodology for data collection, albeit using a different instrument, reaching similar results in the city of Veranópolis (RS), where 57.0% of aged population studied reported significant values of positive satisfaction with life. A study^(^ ^19^ ^)^ conducted with 391 elderly people, aged 64.4-75.1 years, registered at a PCU - in the city of Belo Horizonte (MG), showed 52.4% of sample considered itself satisfied or very satisfied with its current quality of life; of these, 68.6% were satisfied or very satisfied with their health status.

Despite the process of aging not being necessarily related to diseases and disabilties, the chronic-degenerative diseases are frequent among the elderly.^(^ ^22^ ^)^ In this study, a portion of the studied population presented with a health problem (58.5%). Xavier et al.,^(^ ^21^ ^)^ when comparing those satisfied with their quality of health with the dissatisfied, noted that in both groups, there were elderly individuals with health problems, but these were predominantly in the group of those dissatisfied.

It is worth discussing that the type of self-reported chronic disease and its severity can also have a strong influence on the practice of self-medication, but the diseases were not named in this study. Further analyses may be tested using these variables mentioned, or others, in order to verify new associations.

Self-medication was significantly greater in the elderly with chronic diseases and it was mentioned not only in our study. It was also described in the study done by Cascaes et al.,^(^ ^23^ ^)^ carried out with the elderly in the city of Tubarão (SC), where it was noted, especially in situations of pain, that the elderly use medications without medical prescriptions, as well as natural remedies. Some aspects about self-medication were also described in the study by Loyola Filho et al.,^(^ ^24^ ^)^ in which 46% of the aged reported the use of non-prescribed medications in the previous 90 days. This shows how alarming this situation becomes, considering self-medication leads to various risks to the population, especially the elderly, and can even delay an adequate diagnosis and disguise a disease.^(^ ^25^ ^)^

Further studies are required to identify the situation of the aged population in different Brazilian regions, with the purpose of serving as a basis for public health programs geared towards health education and promotion. The ESF should act more effectively in promoting and protecting health, discouraging selfmedication and promoting autonomy in the aged, with consequent satisfaction with their health status.

## CONCLUSION

The presence of at least one chronic disease was reported by most of those interviewed, and the practice of self-medication is significantly more frequent among these individuals. Even so, the degree of satisfaction with one's own health conditions was reported in a predominantly positive manner, both regarding the previous year and compared to other people of the same age.
